# Elucidating Polypharmacological Mechanisms of Polyphenols by Gene Module Profile Analysis

**DOI:** 10.3390/ijms150711245

**Published:** 2014-06-25

**Authors:** Bin Li, Min Xiong, Hong-Yu Zhang

**Affiliations:** National Key Laboratory of Crop Genetic Improvement, Agricultural Bioinformatics Key Laboratory of Hubei Province, Huazhong Agricultural University, Wuhan 430070, China; E-Mails: skylib777@gmail.com (B.L.); mix@stowers.org (M.X.)

**Keywords:** polypharmacology, polyphenol, biclustering analysis, target

## Abstract

Due to the diverse medicinal effects, polyphenols are among the most intensively studied natural products. However, it is a great challenge to elucidate the polypharmacological mechanisms of polyphenols. To address this challenge, we establish a method for identifying multiple targets of chemical agents through analyzing the module profiles of gene expression upon chemical treatments. By using FABIA algorithm, we have performed a biclustering analysis of gene expression profiles derived from Connectivity Map (cMap), and clustered the profiles into 49 gene modules. This allowed us to define a 49 dimensional binary vector to characterize the gene module profiles, by which we can compare the expression profiles for each pair of chemical agents with Tanimoto coefficient. For the agent pairs with similar gene expression profiles, we can predict the target of one agent from the other. Drug target enrichment analysis indicated that this method is efficient to predict the multiple targets of chemical agents. By using this method, we identify 148 targets for 20 polyphenols derived from cMap. A large part of the targets are validated by experimental observations. The results show that the medicinal effects of polyphenols are far beyond their well-known antioxidant activities. This method is also applicable to dissect the polypharmacology of other natural products.

## 1. Introduction

Since reactive oxygen species (ROS), e.g., superoxide radical, hydrogen peroxide, and hydroxyl radical, are involved in the pathogenesis of many diseases, such as cancer, neurodegenerative diseases and atherosclerosis [1], antioxidants in particular polyphenolic antioxidants, have been widely expected to exert prophylactic or therapeutic effects on these diseases [2,3,4,5]. However, a large number of researches indicated that the strong *in vitro* antioxidant activities of polyphenols can not be translated into *in vivo* therapeutic effects [5,6,7,8,9]. This antioxidant paradox was primarily explained by the poor bioavailability of exogenous polyphenols [10]. Our analysis about the biological roles of polyphenols revealed that they were evolved for filtering UV light rather than scavenging intense ROS, which provided an evolutionary explanation to the weak *in vivo* radical-scavenging potential of polyphenols [11]. The evolutionary consideration also suggested that natural polyphenols have evolved an excellent scaffold with well-balanced rigidity and flexibility to adapt to different structures of enzymes in the biosynthetic pipeline, which enables the compounds to bind various proteins [12]. This finding implies that natural polyphenols have inherent potential to exert polypharmacological effects other than redox modulation [13]. However, how to elucidate the polypharmacological mechanisms of natural polyphenols is a great challenge, because the conventional methods to dissect drug mode of action (MoA) are laborious and low throughput [14].

Recently, gene expression-based analysis showed great potential in identifying drug targets [15,16,17]. But the existent methods for gene expression profile analysis normally use limited signature genes (usually corresponding to ~500 probes out of 22,000+), which lose valuable information. In addition, these methods are efficient to reveal a single MoA or target for a certain drug, rather than its polypharmacological mechanisms [16]. Since gene expression signatures related to different biological activities cluster into different modules [18], we speculate that the polypharmacological mechanisms of polyphenols may be better dissected in terms of module profiles of gene expression.

In a previous analysis about connectivity map (cMap), which contains 7056 expression profiles of 5 different human cell lines treated with 1309 agents (including 20 polyphenols), we generated 49 gene modules by using biclustering approach FABIA (factor analysis for bicluster acquisition) [19]. Through analyzing the biological functions of the modules, we revealed that some polyphenols exert polypharmacological effects through activating transcription factors, such as estrogen receptors, nuclear factor (erythroid-derived 2)-like 2, and peroxisome proliferator-activated receptor gamma. In this study, we first establish a gene module-based target identification method and then use this method to further elucidate the polypharmacological mechanisms for the 20 polyphenols.

## 2. Results and Discussion

**Figure 1:**
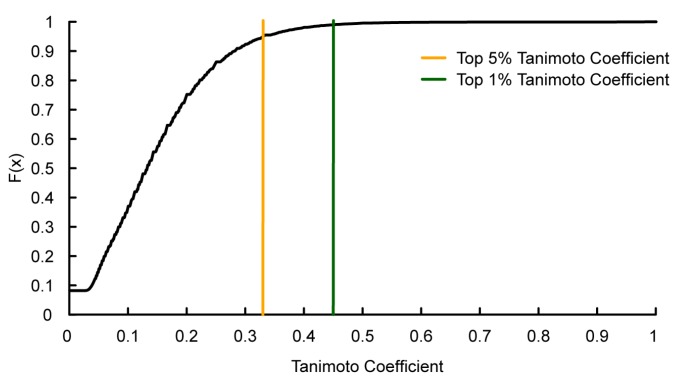
Cumulative frequency (F(*x*)) of pairwise Tanimoto coefficients for 1309 agents.

In a prior research, the cMap-derived 1309 agents and expression profiles have been grouped into 49 gene modules by FABIA algorithm [19], which consist of 5921 probes, much greater than those used in the conventional microarray analysis [15,16]. Thus, each chemical agent in cMap has a gene module profile, which is defined by a 49 dimensional binary vector, with 1 or 0 representing the presence or not of the module (Table S1). This allows us to calculate Tanimoto coefficient for each pair of the compounds to characterize the similarity of their gene expression profiles. The bigger the Tanimoto coefficient is, the more similar biological effects of the compound pairs are expected. For the compound pairs with similar gene module profiles, if one has the MoA and/or target information, we can predict the medicinal behaviors of the other. A total of 856,086 pairwise Tanimoto coefficients were calculated for the 1309 compounds in the cMap dataset (Table S2). The top 1% and 5% coefficients are higher than 0.45 and 0.33, respectively (Figure 1).

To evaluate the effectiveness of this parameter in target identification, we performed a target enrichment test. First, by searching DrugBank [20] and Therapeutic Target Database (TTD) [21], we retrieved 573 approved drugs from 1309 agents, which hit 536 targets. Then, we found that 209 targets were shared by at least two drugs. These targets and corresponding 476 drugs can be used to assess the target enrichment significance. Although the drug targets collected by DrugBank and TTD may be incomplete and may be indirect targets, these information have been successfully used by previous studies to evaluate the target enrichment efficiency [16]. 113,050 pairwise Tanimoto coefficients were calculated for the 476 drugs. The drug pairs with Tanimoto coefficients of higher than 0.33 were used to estimate the probability of target sharing by hypergeometric test. The results showed that 78 targets of 128 drugs can be enriched (*q* < 0.05) (Table S3). It is noteworthy that 96 of 128 drugs have multiple targets (≥2), for which the average ratio of target enrichment reaches 68.75% (66/96) (Table S3). In particular, the 7 targets of chlorpromazine, 8 targets of maprotiline, and 14 targets of imipramine were completely enriched (Table S3). Thus, the present method has great potential to predict MoA and targets of chemical agents, especially to dissect the polypharmacological mechanisms of natural products.

The cMap-derived 1309 agents involve four kinds of polyphenols, *i.e.*, flavonoids (16 agents), monolignols (2 agents) and stilbenoids (1 agent), phenylpropanoids (1 agent). The gene module profiles of these polyphenols show that they are involved in more gene modules than other agents (14.85 ± 4.80 *vs.* 11.85 ± 5.42, *p* < 0.01, *t*-test), suggesting that polyphenols indeed have more complex biological functions than others. The most common modules covered by the 20 polyphenols include module 11 (with occurrence of 14), module 18 (with occurrence of 13), module 25 (with occurrence of 13), module 7 (with occurrence of 12), and module 3 (with occurrence of 12). According to the previously enriched biological functions of 49 gene modules [19], the major functions associated with these modules are protein transport, protein location, cytoskeleton organization, cell motion, purine and pyrimidine metabolism, oxidative phosphorylation, cell cycle, RNA processing, ubiquitin-dependent protein catabolic process and translational elongation. By searching in GeneDecks [22], it was found that four of the five common modules (modules 3, 11, 18 and 25) are tightly linked to cancer and tumors (*p* < 0.0001).

There are 93 drugs that are similar to the 20 polyphenols in terms of gene expression module profile (with Tanimoto coefficients > 0.45), which correspond to 148 targets and provide meaningful clues to clarifying the polypharmacology for these polyphenols (Table S4). In the predicted medicinal effects, anti-neoplastic is most popular (with occurrence of 17 in 93 drugs), in good agreement with the above finding that cancer is linked to most common gene modules.

Table 1, Table 2, Table 3 and Table 4 list the predicted targets of four most intensively studied polyphenols, including genistein (a representative component of soybean), quercetin (one of most widely distributed flavonoids), resveratrol (a representative component of red wine), and (−)-catechin (a representative component of green tea). It can be seen that antineoplastic and antihypertensive are the most common predicted activities of the four polyphenols, which agree well with the health benefits of their dietary sources. For instance, accumulating evidence indicated that high soybean intake and regular green tea drinking are associated with low incidence rates of human cancers and hypertension [23,24,25,26,27,28]. In addition, a large part (50%) of the predicted targets of these polyphenols are validated by experiments, most (92.3%) of which are direct targets (Table 1, Table 2, Table 3 and Table 4). These results strongly warrant the experimental evaluation of other predicted targets.

It is intriguing to note that phosphodiesterase enzymes (PDEs) and estrogen receptor are predicted targets for three of four polyphenols. This finding agrees well with the opinion that plant polyphenols collectively behave as phytoestrogens and can inhibit several isoforms of PDEs [29,30,31]. A major progress in recent natural medicine research was the identification of PDEs as the target of resveratrol [32]. The present analysis highlights the similar pharmacological mechanisms underlying genistein and quercetin.

**Table 1:** Predicted similar drugs and associated targets of genistein.

Drugs	Therapeutic Uses	Targets	References
Imatinib	Antineoplastic Agents	Platelet-derived growth factor receptor ^a^	[33]
		Proto-oncogene tyrosine-protein kinase ABL1 ^a^	[34]
		Mast/stem cell growth factor receptor ^a^	[35]
Raloxifene	Antihypocalcemic Agents	Estrogen receptor ^a^	[36]
Iloprost	Antihypertensive Agents	Prostaglandin E2 receptor, EP2 subtype ^b^	[37]
		cAMP-specific 3',5'-cyclic phosphodiesterase ^a^	[38]
		Prostacyclin receptor ^c^	[37]
Cisapride	Anti-Ulcer Agents	5-Hydroxytryptamine 4 receptor	-
	Gastrointestinal Agents		
	Prokinetic Agents		
Fluticasone	Anti-inflammatory Agents	Glucocorticoid receptor ^a^	[39]
Diethylstilbestrol	Antineoplastic Agents	Estrogen receptor ^a^	[36]
Finasteride	Anti-baldness Agents	Steroid-5-alpha reductase ^a^	[40]
	Antihyperplasia Agents		
Sulindac sulfide	Rheumatoid arthritis	-	-
Prednisone	Anti-inflammatory Agents	Glucocorticoid receptor ^a^	[39]
	Antineoplastic Agents		
Estradiol	Anti-menopausal Agents	Estrogen receptor ^a^	[36]
	Anticholesteremic Agents		
Dydrogesterone	Progesterones	Progesterone receptor	

^a^ as direct targets of genistein; ^b^ as indirect target of genistein which increases prostaglandin release; ^c^ as indirect target of genistein which increases prostacyclin release.

**Table 2:** Predicted similar drugs and associated targets of quercetin.

Drugs	Therapeutic Uses	Targets	References
Tolazoline	Adrenergic alpha-Antagonists	Alpha adrenergic receptor	-
	Antihypertensive Agents		
	Vasodilator Agents		
Tamoxifen	Antineoplastic Agents	Estrogen receptor ^a^	[41]
	Bone Density Conservation Agents		
Finasteride	Anti-baldness Agents	Steroid-5-alpha reductase	-
	Antihyperplasia Agents		
	Skin and Mucous Membrane Agents		
Sulindac sulfide	Rheumatoid arthritis	-	-
Iloprost	Antihypertensive Agents	Prostaglandin E2 receptor, EP2 subtype	-
		cAMP-specific 3',5'-cyclic phosphodiesterase ^a^	[42]
		Prostacyclin receptor	-
Raloxifene	Antihypocalcemic Agents	Estrogen receptor ^a^	[41]
	Bone Density Conservation Agents		
Apomorphine	Antiparkinson Agents	Dopamine receptor ^a^	[43]
		Adrenergic receptors	-
		5-Hydroxytryptamine receptor ^a^	[43]
Fluticasone	Anti-inflammatory Agents	Glucocorticoid receptor	-
Tocainide	Anti-Arrhythmia Agents	Sodium channel protein type 5 subunit alpha ^a^	[44]

^a^ as direct targets of quercetin.

**Table 3:** Predicted similar drugs and associated targets of resveratrol.

Drugs	Therapeutic Uses	Targets	References
Reserpine	Antihypertensive Agents	Synaptic vesicular amine transporter	-
	Antipsychotic Agents		
Mercaptopurine	Antineoplastic Agents	Hypoxanthine-guanine phosphoribosyltransferase	-
	Immunosuppressive Agents		
Niclosamide	Antiparasitic Agents	-	-
Daunorubicin	Antineoplastic Agents	DNA topoisomerase	-
Terfenadine	Anti-Allergic Agents	Histamine H1 receptor	-
	Antiarrhythmic Agents	Potassium voltage-gated channel subfamily H member 2 ^a^	[45]
		Muscarinic acetylcholine receptor M3	-
Fluphenazine	Antipsychotic Agents	Dopamine receptor	-
Dipyridamole	Vasodilator Agents	Adenosine deaminase	-
		cGMP-specific 3',5'-cyclic phosphodiesterase ^a^	[46]
Rescinnamine	Antihypertensive Agents	Angiotensin-converting enzyme ^a^	[47]
Trifluoperazine	Antipsychotic Agents	Dopamine receptor	-
Metixene	Antiparkinson Agents	Muscarinic acetylcholine receptor	-

^a^ as direct targets of resveratrol.

**Table 4:** Predicted similar drugs and associated targets of (−)-catechin.

Drugs	Therapeutic Uses	Targets	References
Letrozole	Antineoplastic Agents	Cytochrome P450 19A1 ^a^	[48]
Triprolidine	Anti-Allergic Agents	Histamine H1 receptor	
Pindolol	Antihypertensive Agents	Adrenergic receptor	-
	Vasodilator Agents	5-hydroxytryptamine receptor	-
Norfloxacin	Anti-Bacterial Agents	DNA topoisomerase 2-alpha ^a^	[48]
Prilocaine	Anesthetics	Sodium channel protein type 5 subunit alpha	-
Estradiol	Anti-menopausal Agents	Estrogen receptor ^a^	[49]
	Anticholesteremic Agents		
Doxycycline	Anti-Bacterial Agents	30S ribosomal protein	-
Bendroflumethiazide	Antihypertensive Agents	Solute carrier family 12 member 3	-
		Calcium-activated potassium channel subunit alpha 1	-
		Carbonic anhydrase	-
Theophylline	Bronchodilator Agents	Adenosine A1 receptor	-
	Vasodilator Agents	cGMP-specific 3',5'-cyclic phosphodiesterase ^a^	[29]
Naltrexone	Anti-craving Agents	Opioid receptor ^a^	[50]

^a^ as direct targets of (−)-catechin.

## 3. Experimental

### 3.1. Tanimoto Coefficient Calculation

Tanimoto coefficient (*TC*) was calculated with a perl program to compare the gene module profiles of each compound pair.

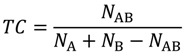
(1)
where *N*_A_ and *N*_B_ are the number of bits set for gene module profiles of compounds A and B, respectively, and *N*_AB_ is the set bits that A and B have in common. If *TC* = 1, the compound pair have the same module profiles; if *TC* = 0, the pair have totally different module profiles. 

### 3.2. Drug Target Enrichment

Hypergeometric test was used to assess the drug target enrichment significance. The Equation (2) was derived by computing the extreme tail probabilities:

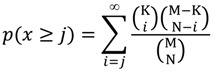
(2)
where N is the number of total approved drugs for target enrichment (*i.e.*, 476), M is the number of drugs involving the similar module profiles (with Tanimoto coefficient > 0.33), *i* is the number of drugs sharing the same target in N, K is the number of drugs sharing the same target in M. Thus, we can calculate the probability by chance, at least *x* occurrences of a target among those associated with the M drugs. The *p*-values were further adjusted by False Discovery Rate calculation (with R function ‘p.adjust()’ using Benjamini-Hochberg method [51]). The enriched targets were ranked by *p*-value from most significant to least significant. Then, for each target the *q*-value is calculated by Equation (3):

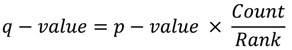
(3)
where *Count* is the total number of enriched targets. The enriched targets were then selected using a *q*-value threshold of 0.05.

## 4. Conclusions

Natural products (NPs) have made important contributions to safe guarding human health. Not only ancient humans depended on NPs to cure various diseases, modern pharmaceutical industry also benefit from NPs to find hits, leads and drugs [12]. Therefore, it is of great significance to elucidate the therapeutic mechanisms of NPs. However, this is a big challenge, because NPs usually hit multiple targets with relatively weak affinity and the conventional target identification methods are laborious and low throughput [14].

In this study, we established a gene module-based target identification method. Because gene modules cover more gene probes, this method is more efficient than conventional microarray analysis methods in information extraction. Therefore, this method enables the discovery of richer information about the medicinal effects of chemical agents, which is very helpful to clarify the polypharmacological mechanisms of polyphenols and other NPs. Moreover, this method may be used to predict targets for NPs beyond those contained in cMap, so it is expected to find more and more applications in the omics era, because the NP-related microarray data are rapidly accumulated.

## Supplementary Files

Supplementary File 1
